# An innovative deformation coordination method for analyzing distortion effects on box girders

**DOI:** 10.1038/s41598-024-69130-y

**Published:** 2024-08-27

**Authors:** Chenguang Wang, Mingxin Shi, Jianqiang Huang, Yuanhai Zhang, Weiwen Li, Walid Mansour, Linyuwen Ke, Peng Wang

**Affiliations:** 1 School of Civil Engineering, Lanzhou Jiaotong University, Lanzhou, 730070 China; 2 Guangdong Provincial Key Laboratory of Durability for Marine Civil Engineering, Shenzhen University, Shenzhen, China; 3 Department of Civil and Environmental Engineering, The Hong Kong Polytechnic University, Hong Kong, China; 4 Civil Engineering Department, Faculty of Engineering, Kafrelsheikh University, Kafrelsheikh, Egypt; 5 Department of Hong, Kong University of Science and Technology, Hong Kong, China

**Keywords:** Box girder, Distortion effects, Deformation coordination method, Plate element analysis method, Total potential energy variation method, Civil engineering, Applied mathematics

## Abstract

A deformation coordination method is proposed in this study to account for the distortion effects on a box girder. The differential equation for distortion in vertical web box girders is derived based on the deformation coordination condition of the distortion angle, considering both external loads and internal forces. Subsequently, a comparative analysis is conducted to explore the similarities and differences between the differential equations derived from the proposed deformation coordination method, the plate element analysis method and the total potential energy variation method. The accuracy of the proposed approach is verified through bench-scale tests and numerical simulations. The findings indicate that the derived governing distortion differential equation and distortion attenuation coefficients in the proposed method align with those obtained from the plate element analysis method and the total potential energy variational method, which enhances the applicability to allow for the distortion equations to be obtained simply by calculating the distortion displacements. The analytical findings regarding the distortion warping normal stresses on the cross-sections of the box girders demonstrate favorable correspondence with the experimental results, displaying an acceptable error ranging from − 0.3% to 5.4%. Moreover, the peak of distortion warping normal stresses on the mid-span cross-section increases with higher span-to-depth ratios and height-to-thickness ratios of the web. Consequently, augmenting the thickness of the box wall proves to be an effective means of reducing the distortion effect in box girders.

## Introduction

Thin-walled single-cell box beams have gained widespread acceptance in the construction of medium- and long-span highway bridges^1^, primarily due to their visual aesthetic and exceptional resistance to bending and torsional forces. However, when subjected to torsional loading, the cross-section of a thin-walled box beam may suffer from distortion, which primarily results in warping stresses. These warping stresses can be comparable in magnitude to longitudinal bending stresses, especially in the absence or insufficient rigidity of transverse diaphragms. Therefore, in the transition towards lightweight, thin-walled structures with larger spans, wider rib spacing and reduced transverse diaphragms, it becomes crucial to consider the potential occurrence of distortional behavior, in addition to accounting for bending and torsional effects^2–4^.

Numerous research efforts have been undertaken to investigate the impact of distortion on box girders comprehensively using both analytical and numerical methods. A significant advancement in addressing the general solution of the problem was made through the introduction of the generalized coordinate method^5^. Building upon the generalized coordinate method, Razaqpur and Li^6,7^ and Maisel^8^ introduced an orthogonalization procedure for addressing distortional modes and shear lag modes in the formulation of box beam elements. Schart^9–11^ developed an advanced formulation referred to as Generalized Beam Theory (GBT), which extended the classical Vlasov beam theory to incorporate flexural and torsional distortion. Also, Jonsson and Andreassen^12,13^ established a comprehensive set of deformation modes using eigenvalue-type cross-sectional analysis and then proposed an analytical solution of beam equations to formulate the semi-discretized thin-walled beam element under distortional effects.

The finite element (FE) modeling approach is also utilized to undertake a comprehensive investigation into the effects of distortion. Boswell^14–17^ proposed an FE model for thin-walled box beams with variable cross-sections and then experimentally validated the model’s correctness. Li^18^ developed a one-dimensional beam element with four degrees of freedom (DOF) to study the influence of distortion on thin-walled multi-cellular beams with cantilevered flanges. This approach unifies the displacement components around the cross-section’s edge concerning the distortional center. Zhu et al.^19^ introduced a one-dimensional model (26 DOFs) for curved composite box beams, considering the actual issues such as constrained torsion, distortion, shear lag, biaxial slip at the interface and curvature differences along the width of the beam.

Based on the abovementioned analytical and numerical approaches, the actual distortion issue can be further simplified under the assumption of independent distortion and torsion behaviors, i.e., it is assumed that there is no interaction between these effects^20–22^. Xu et al.^23^ employed the Hellinger–Reissner variational principle to incorporate distortional shear deformation effects, utilizing the first derivative of the distortion angle as the distortional warping function for conventional hollow sections in bridge structures. The research outcome indicated that the distortional shear deformation effects can be neglected. A similar conclusion was obtained by Zhao et al.^24^, confirming the limited influence of the coupling between torsion and distortion in box beam bridges. This observation contributes to the understanding of eccentric load effects in such structures.

Further, the theorem of the total potential energy variational method and the analysis of plate elements are commonly employed to establish the governing equilibrium equations based on the uncoupling assumption. Based on the Newmark method from the conjugate beam theory and incorporated fundamental principles of plate element analysis, Li et al.^25^ developed a distortion calculation method for variable cross-section corrugated steel web composite box beams. Deng et al.^26^ utilized the total potential energy variational method and derived differential equations for the distortion in single-box three-cell cantilever girders with corrugated steel webs. Although the total potential energy variation method and the plate element analysis method are mature techniques for analyzing distortion effects in box girders, they both exhibit certain limitations. Specially, the total potential energy variation method primarily emphasizes the ultimate state of distortion deformation and derives the distortion control differential equation using energy principles. However, this method does not provide insights into the underlying mechanism through which box girders undergo distortion when subjected to loading conditions. The plate element analysis method establishes the distortion control differential equation by effectively balancing internal and external distortion forces. Nevertheless, it does not elucidate the intricate relationship between generalized distortion forces and distortion displacements.

This study introduces a method analyzing the distortion effect of vertical web plate girders, which utilizes the coordination condition of the deformations caused by distortion-induced warping normal stresses, distortion-induced warping shear stresses, and externally induced distortion moments to comprehensively analyze the distortion effects in box girders. In contrast to the two existing methods, the approach proposed in this study offers enhanced clarity regarding its physical interpretation in terms of distortion deformation. The new approach enhances the applicability to allow for the distortion equations to be obtained simply by calculating the distortion displacements. A comparative analysis is conducted to discern the inherent disparities and fundamental correlations between the proposed method and the two existing methods, establishing their consistency in evaluating distortion effects. This congruity substantiates the accuracy and validity of the proposed method, thereby affirming its suitability for practical engineering applications, akin to its existing counterparts^24–27^. Additionally, the research investigates the impact of variations in geometric parameters on the distortion effects observed in box girders. By establishing this framework, a comprehensive understanding of the distortional behavior of box girders can be achieved.

## Distortion deformation and distortion internal forces

**Figure 1:**
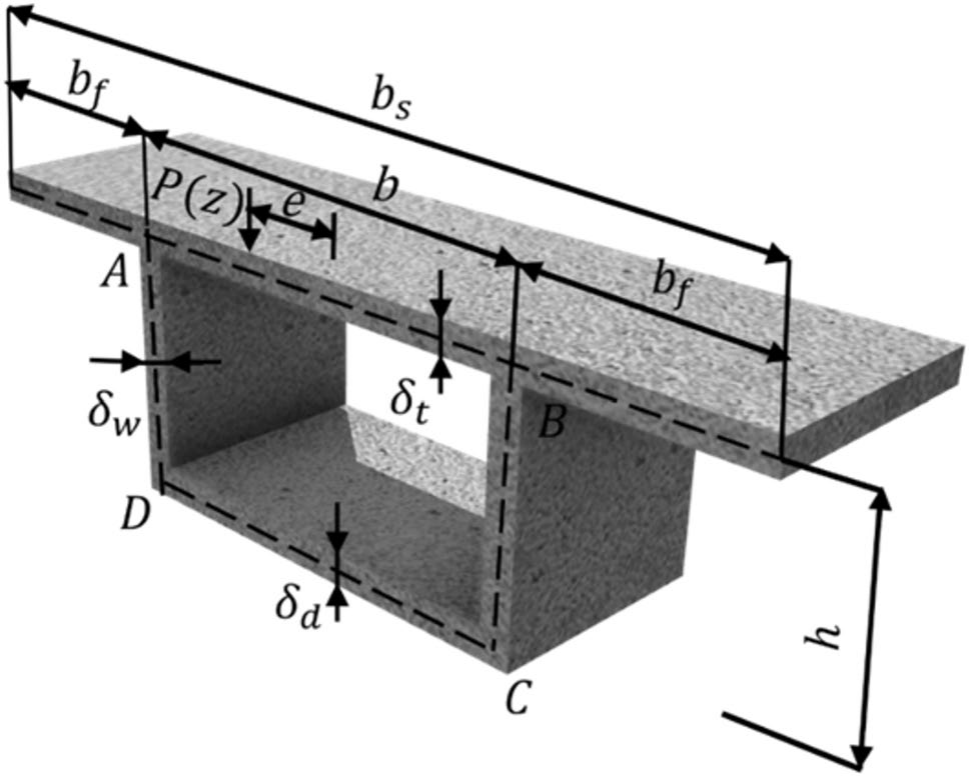
Cross-section of a box girder.

The cross-section of the box girder is schematically shown in Fig. 1, where *b* denotes the width of the bottom plates, *h* denotes the height of the girder, *b*_f_ denotes the flange width, *b*_s_ denotes the width of the top plate, and *δ*_t_, *δ*_d_, and *δ*_w_ denote the thicknesses of the top, bottom and web plates, respectively. Moreover, *e* implies the eccentricity of load *P*(z). The points *A*, *B*, *C* and *D* correspond to the intersections of the top and bottom plates with the web plate.

**Figure 2:**
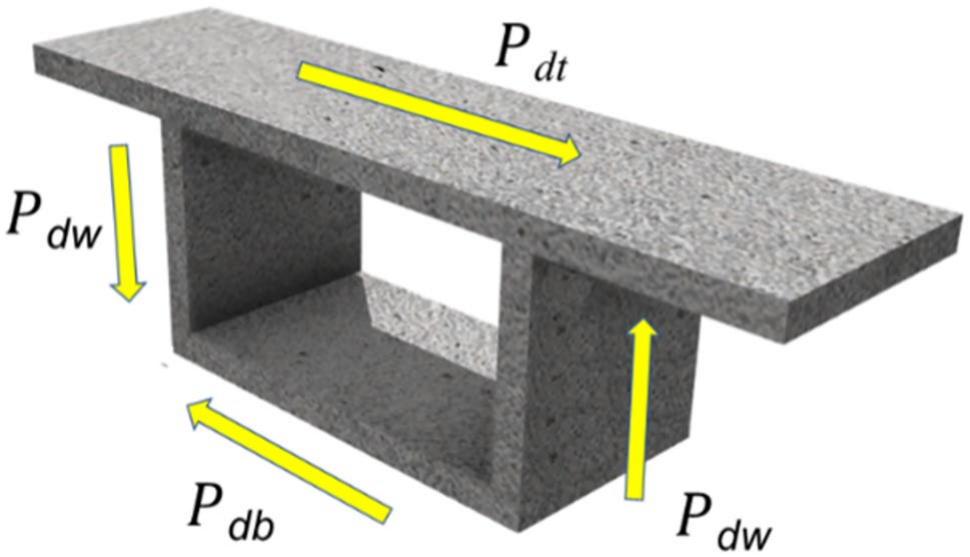
Distortion load on the cross-section of box girder.

The eccentric load *P*(z) can be decomposed into symmetric bending loads, rigid torsion loads and distortion loads. In particular, it is noteworthy that bending loads and rigid torsion loads do not induce distortion deformation in the cross-section. Hence, in the analysis of distortion effects, it is sufficient to consider only the influence of distortion loads. The distortion loads, denoted as *P*_dt_, *P*_db_ and *P*_dw_, acting on the top plate, bottom plate and web plate as illustrated in Fig. 2, are defined by Eq. 1^22^:

1 $$P_{{{\text{dt}}}} { = }\frac{{P_{{\text{v}}} b}}{2h},P_{{{\text{db}}}} { = }\frac{{P_{{\text{v}}} b}}{2h},P_{{{\text{dw}}}} { = }\frac{{P_{{\text{v}}} }}{2}$$

where $P_{{\text{v}}} = \frac{Pe}{b}$ denotes the load that has been transformed or converted.

**Figure 3:**
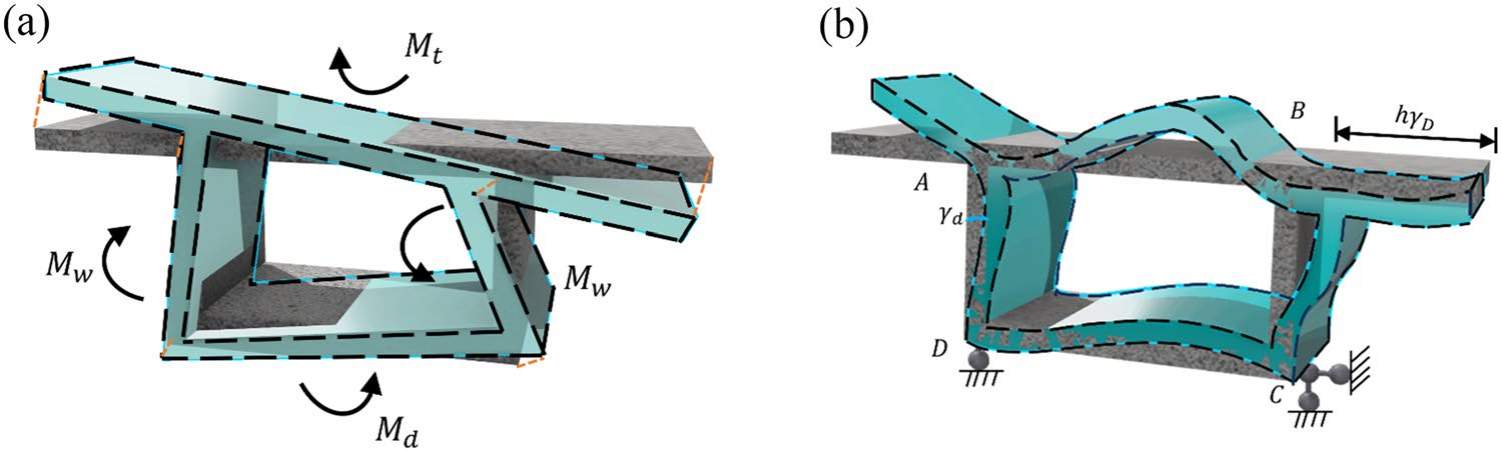
Distortion deformations of a box girder. (**a**) Distortion warping deformation (**b**) Distortion frame deformation.

Under the action of distortion loads, the individual components of the box girder experience distortion warping deformation within their respective planes, as well as lateral frame deformation outside their planes. When analyzing distortion effects in box girders, the change in the angle ∠*ADC* (denoted as ${\upgamma }_{\text{D}}$) between the web plate and bottom plate under the influence of distortion loads is often chosen as the fundamental unknown distortion quantity. It is pertinent to acknowledge that ${\upgamma }_{\text{D}}$ is dependent on distortion warping deformation, distortion frame deformation, and external distortion moment. Ultimately, this leads to the establishment of distortion control differential equations for box girders based on distortion warping shear flow. The various distortion deformations of the box girder are depicted in Fig. 3.

When the box girder undergoes distortion warping deformation as depicted in Fig. 3a, it is assumed that the distortion warping normal stress exhibits a linear distribution across the cross-section. Consequently, distortion warping normal stresses, denoted as *M*_t_, *M*_d_, and *M*_w_ arise in the top plate, bottom plate, and web plate, respectively. Based on the self-balancing condition of distortion warping normal stresses, the following relationships are established:

2 $$M_{{\text{t}}} { = }\frac{{{2}\beta hJ_{{\text{t}}} }}{{\left( {{1 + }\beta } \right)bJ_{{\text{w}}} }} \cdot M_{{\text{w}}}$$

3 $$M_{{\text{d}}} { = }\frac{{{2}hJ_{{\text{d}}} }}{{\left( {{1 + }\beta } \right)bJ_{{\text{w}}} }} \cdot M_{{\text{w}}}$$

where $J_{t} = \frac{{\delta_t b_{s}^{3} }}{12}$, $J_{d} = \frac{{\delta_d b_{d}^{3} }}{12}$,$J_{w} = \frac{{\delta_w b_{w}^{3} }}{12}$;  *β* represents the ratio of distortion warping curvature at the corners (e.g., $\tilde{\omega }_{B}$ and $\tilde{\omega }_{C}$) to the web plate’s thickness, and it can be determined from the self-balancing condition of distortion warping normal stresses:

4 $$\beta { = }\frac{{\tilde{\omega }_{B} }}{{\tilde{\omega }_{C} }}{ = }\frac{{A_{{\text{d}}} + 3A_{{\text{w}}} }}{{A_{{\text{t}}} \left( {1 + \frac{{2b_{{\text{f}}} }}{b}} \right)^{2} + 3A_{{\text{w}}} }}$$

where *A*_t_ is the top plate area, *A*_d_ is the bottom plate area, and *A*_w_ is the web plate area.

The distortion angle *γ*_D_, arising from the bending moments within the planes of the top plate (*M*_t_), bottom plate (*M*_d_), and web plate (*M*_w_), can be determined through the utilization of a plate-beam hinge model. By considering the assumptions of cross-sectional equilibrium and the geometric interdependencies among displacements of different plate elements, the correlation between *γ*_D_ and the bending moment *M*_w_ generated by distortion warping normal stress on the web plate can be formulated as follows:

5 $$\gamma^{\prime\prime}_{D} = \frac{ - 4}{{EJ_{{\text{w}}} b}} \cdot M_{{\text{w}}}$$

where *E* is the elastic modulus.

When the box girder undergoes distortion frame deformation as depicted in Fig. 3b, a model is proposed to establish the relationship between the distortion angle *γ*_D_ and the transverse bending moments at specific corner points within a slender frame of unit length, subjected to a horizontal displacement *hγ*_D_ at the top plate. The lateral bending moments *m*_AB_ and *m*_DC_ at nodes *A* and *D* can be determined as follows:

6 $$m_{{{\text{AB}}}} = \frac{{X_{1} bh}}{{2\delta_{{\text{h}}} }}\gamma_{{\text{D}}} { = }K_{1} \gamma_{{\text{D}}}$$

7 $$m_{{{\text{DC}}}} = \frac{{2h^{2} - X_{1} bh}}{{2\delta_{{\text{h}}} }}\gamma_{{\text{D}}} = K_{2} \gamma_{{\text{D}}}$$

where $K_2 = \frac{{\ 2h^2 -  X_{1} bh}}{2 \delta_h}$, $K_1 = \frac {{X_{1} bh}}{2 \delta_h}$,  *X*_1_ and *δ*_h_ represent the shear force and lateral displacement at the midspan of the top plate when it is subjected to a unit horizontal force.

8 $$X_{1} = 2\frac{{\frac{bh}{{I_{2} }} + \frac{{3h^{2} }}{{I_{1} }}}}{{\frac{{b^{2} }}{{I_{4} }} + \frac{{b^{2} }}{{I_{2} }} + \frac{{6hb^{{}} }}{{I_{1} }}}}$$

9 $$\delta_{{\text{h}}} = \frac{{1}}{{{6}E}}\left[ {\frac{{b^{3} X^{2} }}{{I_{4} }} + \frac{{b(bX - h)^{2} }}{{I_{2} }}{ + }} \right.\left. {\frac{2h}{{I_{1} }}\left( {3b^{2} X^{2} - 3bhX + h^{2} } \right)} \right]$$

where *X* = *X*_1_/2, $I_{1} = \frac{{\delta_{{\text{w}}}^{3} }}{12}$, $I_{2} = \frac{{\delta_{{\text{d}}}^{3} }}{12}$, $I_{4} = \frac{{\delta_{{\text{t}}}^{3} }}{12}$ represent the structural resistance to transverse bending of the web, bottom plate, top plate and flange, respectively.

Upon determining the distortion bending moments affecting the cross-section of the box girder, it becomes possible to evaluate the girder's capacity to resist distortion frames. By utilizing Eqs. 6 and 7, the values of the distortion shear forces *Q*_dt_, *Q*_db_ and *Q*_dw_ exerted on the web plate, bottom plate and top plate, respectively, due to the presence of the distortion frame, can be expressed as follows:

10 $$Q_{{{\text{dt}}}} = Q_{{{\text{db}}}} = \frac{{{2}\left( {m_{{{\text{AB}}}} { + }m_{{{\text{DC}}}} } \right)}}{h}$$

11 $$Q_{{{\text{dw}}}} = \frac{{{2}\left( {m_{{{\text{AB}}}} { + }m_{{{\text{DC}}}} } \right)}}{b}$$

Under internal shear forces *Q*_dt_, *Q*_db_ and *Q*_dw_, a pair of self-equilibrating distortion moments *M*_γ_ is formed on the thin plate frame:

12 $$M_{\gamma } = Q_{{{\text{dw}}}} b = Q_{{{\text{dt}}}} h$$

Substituting Eqs. 6, 7, 10 and Eqs. 11 into Eq. 12, the following Eq. 13 can be obtained:

13 $$M_{\gamma } = K_{{\text{d}}} \gamma_{{\text{D}}}$$

where $K_{{\text{d}}} = \frac{{{24}EI_{1} }}{{h\zeta_{0} }}$ represents the stiffness of the distortion-resistant frame, indicating the distortion moment required to generate a unit distortion angle in the box-beam thin plate frame, $\zeta_{0} = {1 + }\frac{{{2}\frac{b}{h} + 3\frac{{I_{2} + I_{4} }}{{I_{1} }}}}{{\frac{{I_{2} + I_{4} }}{{I_{1} }} + 6\frac{{hI_{2} I_{4} }}{{bI_{1}^{2} }}}}$ denotes a parameter associated with the geometric characteristics of the box beam.

## Deformation coordination method

**Figure 4:**
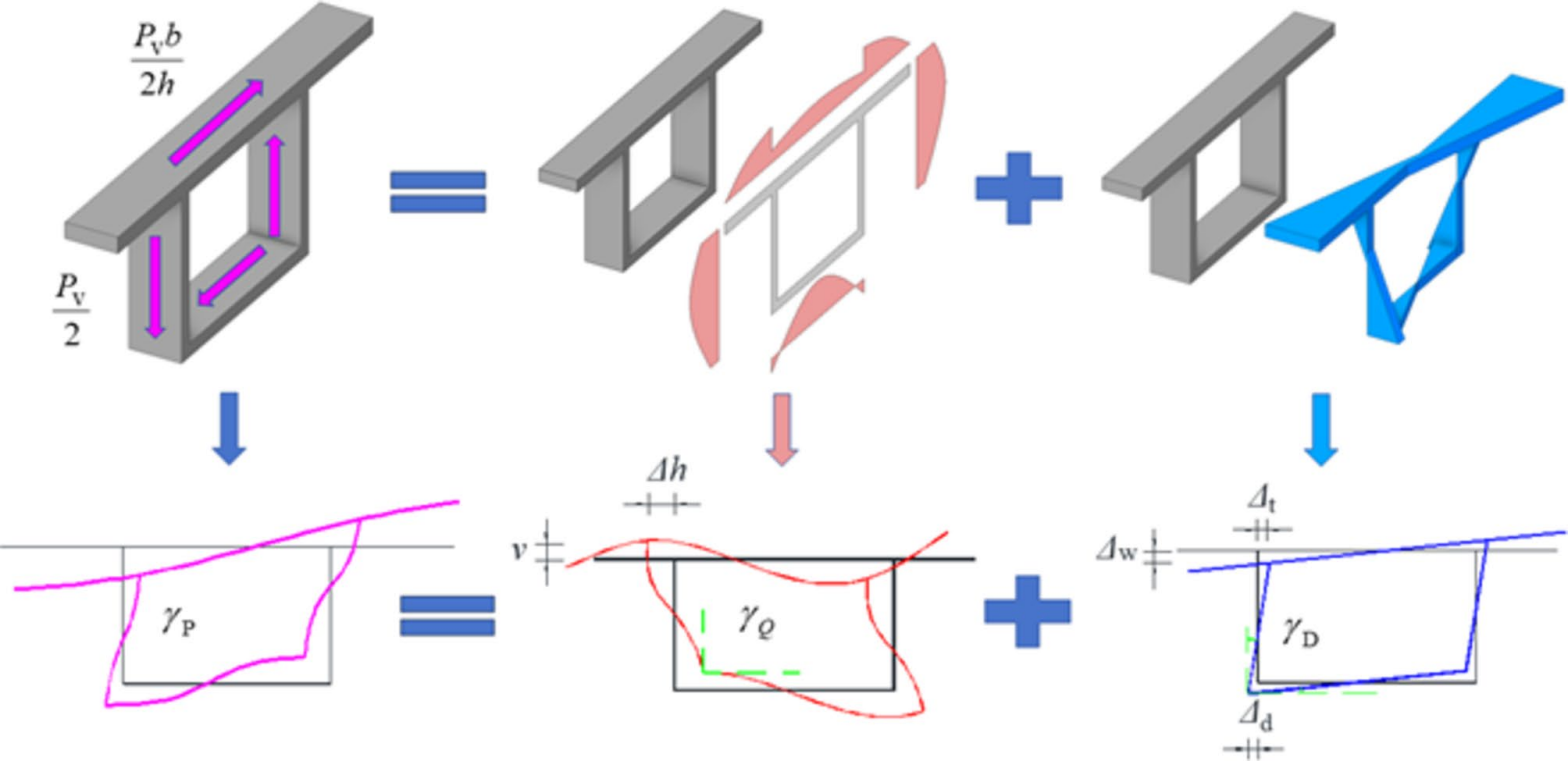
Schematic diagram of the decomposition of distortion displacements.

Figure 4 is a schematic diagram of the decomposition of distortion displacements. In the computation of distortion effects in a box beam employing the distortion angle deformation coordination method, a differential equation governing distortion control is formulated by establishing the deformation coordination relationship between distortion bending normal stress, distortion bending shear stress, and distortion angles induced by external distortion loads. This method enables a comprehensive consideration of the impacts of distortion bending normal stress and distortion bending shear stress throughout the analysis procedure, and it exhibits a well-defined physical concept.

The distortion bending normal stress ${\sigma }_{WD}$ exerted on the cross-section of the box beam can be expressed in generalized coordinates as follows:

14 $$\sigma_{{{\text{WD}}}} { = }f\left( z \right)\tilde{\omega }\left( s \right)$$

where *f*(z) is the generalized displacement, and $\widetilde{\omega }\left(s\right)$ is the generalized coordinate for distortion bending.

By considering the longitudinal equilibrium relationship between distortion bending normal stress and distortion bending shear stress on the elemental box wall, along with the constraint that distortion bending shear flow does not induce torsion at the cross-section, the expression for distortion bending shear flow ${q}_{WD}$ can be formulated as follows:

15 $$q_{{{\text{WD}}}} = - f^{\prime} \cdot \tilde{S}_{{{\text{WD}}}}$$

where ${f}{\prime}$ is the derivative of *f*(z), $\overline{S}_{{{\text{WD}}}} = S_{{{\text{WD}}}} - \frac{1}{2bh}\int_{A} {S_{{{\text{WD}}}} \rho {\text{d}}s}$ is the generalized distortion warping static moment of the box beam, and $S_{{{\text{WD}}}} { = }\int_{{0}}^{s} {\tilde{\omega }t{\text{d}}s}$ is the distortion warping static moment of the box beam. The distribution of the generalized distortion warping static moment $\bar{S}_{{{\text{WD}}}}$ is shown in Fig. 4.

The horizontal distortion shear force *H'*_d_ acting on the top and bottom plates, as well as the vertical distortion shear force *V*'_d_ on the web plate, exhibit a state of self-equilibrium within the thin plate frame, thereby giving rise to the distortion moment *M*_γQ_:

16 $$M_{\gamma Q} = V^{\prime}_{d} b = - f^{\prime\prime}\frac{{bh\tilde{\omega }_{B} }}{12}\left( {\frac{2 - \beta }{\beta }A_{w} + \frac{1}{\beta }A_{d} } \right)$$

Based on Eq. 13, the distortion angle *γ*_Q_ produced by the distortion moment *M*_γQ_ is given by:

17 $$\gamma_{{\text{Q}}} = - \frac{W}{{K_{{\text{d}}} }}f^{\prime\prime}$$

where ${f}^{{\prime}{\prime}}$ is the 2nd-order derivative of *f*(z), $W = \frac{{bh\tilde{\omega }_{B} }}{12}\left( {\frac{2 - \beta }{\beta }A_{{\text{w}}} + \frac{1}{\beta }A_{{\text{d}}} } \right)$ is the geometric characteristic parameter of the cross-section of the box beam.

The distortion bending normal stress, as defined by Eq. 14, results in the generation of the distortion bending moment ${M}_{w}$ on the web plate:

18 $$M_{{\text{w}}} = \frac{{\left( {1 + \beta } \right)J_{{\text{w}}} }}{\beta h}f\tilde{\omega }_{B}$$

Substituting Eqs. 18 into 5, the distortion angle *γ*_D_ satisfies the following condition:

19 $$\gamma^{\prime\prime}_{{\text{D}}} = - \frac{f}{E}$$

The self-equilibrated distortion external load (Fig. 2) gives rise to the distortion moment *M*_γp_ on the thin plate frame of the box beam, with its value equal to $M_{{\gamma {\text{P}}}} = P_{v} b/2$. Subsequently, the induced distortion angle *γ*_p_ can be calculated based on Eq. 13:

20 $$\gamma_{{\text{P}}} = \frac{{P_{{\text{v}}} b}}{{2K_{{\text{d}}} }}$$

Based on the deformation coordination condition of distortion angles, i.e., the distortion angle generated by the box beam under external distortion loads is equal to the distortion angle jointly produced by distortion bending normal stress and distortion bending shear stress, the distortion angle deformation coordination equation is established as follows:

21 $$\gamma_{{\text{P}}} = \gamma_{{\text{D}}} + \gamma_{{\text{Q}}}$$

Substituting Eqs. 17, 19 and 20 into Eq. 21, the governing distortion differential equation for the box beam is obtained based on the deformation coordination method:

22 $$\gamma_{{\text{D}}}^{\prime \prime \prime \prime } { + 4}\lambda_{{\text{S}}}^{4} \gamma_{{\text{D}}} { = }\frac{{\tilde{M}_{{{\text{DS}}}} }}{{EI_{{{\text{DS}}}} }}$$

where the distortion attenuation coefficients ($\lambda_{{\text{S}}} { = }\sqrt[{4}]{{\frac{{EI_{{{\text{WS}}}} }}{{{4}EI_{{{\text{DS}}}} }}}}$), distortion framework stiffness ($EI_{{{\text{WS}}}} = \frac{{{24}EI_{1} }}{{h\zeta_{0} }}$), external load distortion moment ($\tilde{M}_{{{\text{DS}}}} = \frac{{P_{{\text{v}}} b}}{2}$) and torsional stiffness against distortion ($EI_{{{\text{DS}}}} = \frac{{Eb^{2} h^{2} }}{{{48}}}\frac{{\left( {2 - \beta } \right)A_{{\text{w}}} + A_{{\text{d}}} }}{1 + \beta }$) are computed in accordance with the deformation coordination method.

## Plate element analysis method

When analyzing the distortion effects of a box beam using the plate element method, the various plate components that make up the box beam are discretized into plate elements. The forces corresponding to lateral bending distortion are defined as the in-plane external force systems of each plate element, while the forces corresponding to torsional distortion are defined as the in-plane internal force systems of each plate element. The relationship between distortion deformation under distortion loads and torsional deformation is determined through the balance conditions of the in-plane internal force systems. Subsequently, the governing differential equations for the distortion of box beam are derived through supplementary balance conditions of the in-plane external force systems.

**Figure 5:**
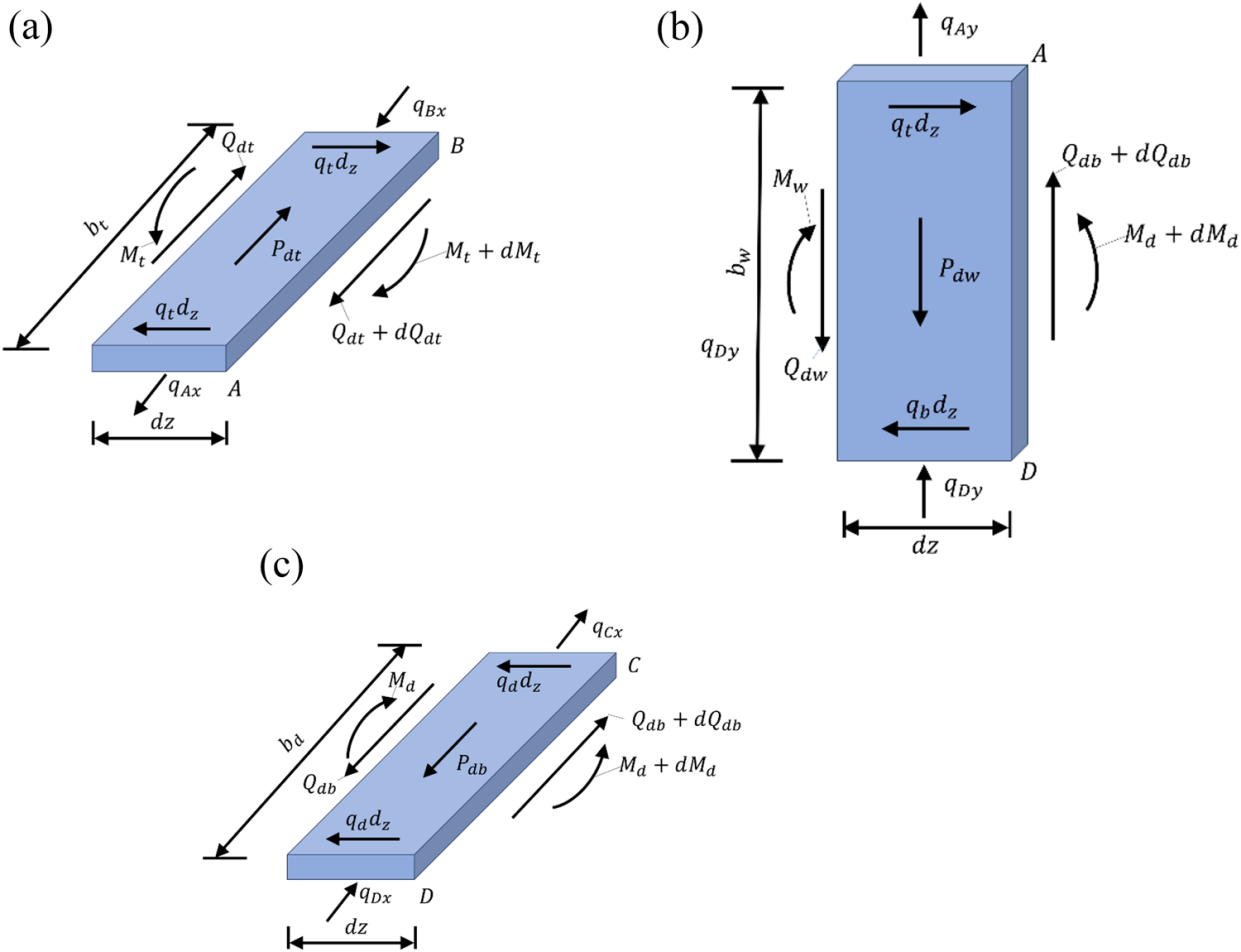
The in-plane force system of the plate element. (**a**) Top plate (**b**) Web plate (**c**) Bottom plate.

The in-plane internal force systems acting on each plate element are shown in Fig. 5, where $q_{{\text{t}}}$ and $q_{{\text{d}}}$ respectively represent the longitudinal restraining forces exerted by the web plate elements against the top and bottom plates, $q_{{{\text{Ax}}}}$ and $q_{{{\text{Bx}}}}$ represent the lateral restraining forces exerted by the left and right-side web plate elements on the top plate elements, $q_{{{\text{Cx}}}}$ and $q_{{{\text{Dx}}}}$ represent the lateral restraining forces exerted by the left and right-side web plate elements on the bottom plate elements, $q_{{{\text{Ay}}}}$ and $q_{{{\text{Dy}}}}$ represent the vertical restraining forces exerted by the top and bottom plate elements on the left-side web plate element, and $Q_{{{\text{dt}}}}$, $Q_{{{\text{db}}}}$ and $Q_{{{\text{dw}}}}$ represent the shear forces acting on the top plate element, bottom plate element, and web plate element, respectively.

By considering the equilibrium of forces among the plate elements, the following relationships for the distortion and deformation of a box girder can be established:

23 $$W_{3} = W_{2} - W_{1}$$

where *W*_1_, *W*_2_ and *W*_3_ are quantities related to distortion warping deformation, distortion frame deformation and distortion external loads, respectively:

24 $$W_{1} = \frac{{{\text{d}}^{2} M_{{\text{w}}} }}{{{\text{d}}z^{2} }} + \frac{{b_{{\text{w}}} }}{{{2}b_{{\text{t}}} }}\frac{{{\text{d}}^{2} M_{{\text{t}}} }}{{{\text{d}}z^{2} }} + \frac{{b_{{\text{w}}} }}{{{2}b_{{\text{d}}} }}\frac{{{\text{d}}^{2} M_{{\text{b}}} }}{{{\text{d}}z^{2} }}$$

25 $$W_{{2}} = Q_{{{\text{dw}}}} + \frac{{b_{{\text{w}}} }}{{2b_{{\text{t}}} }}Q_{{{\text{dt}}}} + \frac{{b_{{\text{w}}} }}{{2b_{{\text{d}}} }}Q_{{{\text{db}}}}$$

26 $$W_{{3}} = \frac{{b_{{\text{w}}} }}{{{2}b_{{\text{t}}} }}P_{{{\text{dt}}}} { + }\frac{{b_{{\text{w}}} }}{{{2}b_{{\text{d}}} }}P_{{{\text{db}}}} + P_{{{\text{dw}}}}$$

It is noteworthy that Eq. 21 demonstrates the deformation coordination relationship among distortion displacements, while Eq. 23 illustrates the equilibrium relationship between distortion internal forces and distortion external loads.

Substituting Eqs. 2 and 3 into Eq. 24, Eqs. 10 and 11 into Eqs. 25, and 1 into Eq. 26, *W*_1_, *W*_2_ and *W*_3_ can be further expressed as:

27 $$W_{1} = \frac{{\Gamma_{{1}} }}{{\Gamma_{{2}} }}\gamma_{{\text{D}}}^{\prime \prime \prime \prime }$$

28 $$W_{{2}} = \frac{{4h^{2} }}{{b\delta_{{\text{h}}} }}\gamma_{{\text{D}}}$$

29 $$W_{3} { = }P_{{\text{v}}}$$

where $$\Gamma_{{1}} = 1 + \frac{{h^{2} \left( {\beta J_{{\text{t}}} + J_{{\text{d}}} } \right)}}{{\left( {{1 + }\beta } \right)b^{2} J_{{\text{w}}} }}$$ and $$\Gamma_{{2}} { = }\frac{ - 4}{{EJ_{{\text{w}}} b}}$$.

Substituting **Eqs. **27, 28 and 29 into **Eq. **23, the governing distortion differential equation for a box girder can be obtained based on the plate element method:

30 $$\gamma_{{\text{D}}}^{\prime \prime \prime \prime } + 4\lambda_{{\text{P}}}^{4} \gamma_{{\text{D}}} = \frac{{\tilde{M}_{{{\text{DP}}}} }}{{EI_{{{\text{DP}}}} }}$$

where $\lambda_{{\text{P}}} { = }\sqrt[{4}]{{\frac{{EI_{{{\text{WP}}}} }}{{{4}EI_{{{\text{DP}}}} }}}}$ is the distortion attenuation coefficients, $EI_{{{\text{WP}}}} = \frac{{4h^{2} }}{{\delta_{{\text{h}}} }}$ is distortion frame stiffness,$\tilde{M}_{{{\text{DP}}}} = P_{{\text{v}}} b$ is the distortion external moment and $EI_{{{\text{DP}}}} = - \frac{{\Gamma_{1} b}}{{\Gamma_{2} }}$ is the distortion warping stiffness of the box girder.

## Total potential energy variational method

By considering the distortion angle as the primary unknown in distortion displacement, the governing distortion differential equation for the box girder can be derived by evaluating the distortion frame strain energy (*U*_1_), distortion warping strain energy (*U*_2_) and external load potential energy (*V*) of the box girder when distortion deformation takes place. This derivation follows the principle of minimum potential energy.

Based on **Eqs. **6 and 7, the distortion frame strain energy *U*_1_ can be computed as^24^:

31 $$U_{{1}} = \int_{0}^{l} {\int_{s} {\frac{{M^{2} }}{2EI}{\text{d}}s{\text{dz}}} } = K_{3} \int_{0}^{l} {\gamma_{{\text{D}}}^{2} {\text{d}}z}$$

$$K_{3} = \frac{1}{6E}\left[ {K_{1}^{2} \frac{b}{{I_{4} }} + K_{2}^{2} \frac{b}{{I_{2} }} + \frac{{2h\left( {K_{1}^{2} + K_{2}^{2} - K_{1} K_{2} } \right)}}{{I_{1} }}} \right]$$.

where the distortion warping internal moment *M*_w_ acting on the web plate is expressed in terms of the distortion warping normal stress ${\sigma }_{D}$ at point *D*, as obtained from Eq. (5):

32 $$\sigma_{{\text{D}}} { = }EK_{4} \gamma^{\prime\prime}_{{\text{D}}}$$

$$K_{4} { = }\frac{bh}{{4\left( {1 + \beta } \right)}}$$.

where the distortion warping normal stress $\sigma_{{{\text{WD}}}}$ is linearly distributed across the cross-section, so the distortion warping normal stress at any point can be obtained from the distortion warping normal stress ${\sigma }_{D}$ at point *D*. Thus, the distortion warping strain energy *U*_2_ is given by^24^

33 $$U_{{2}} = \int_{0}^{l} {\int_{A} {\frac{{\sigma_{{{\text{WD}}}}^{2} \left( {z,s} \right)}}{2E}} } {\text{d}}A{\text{d}}z = H\int_{0}^{l} {\left( {\gamma^{\prime\prime}_{{\text{D}}} } \right)}^{2} {\text{d}}z$$

where

34 $$H = \frac{{EK_{4}^{2} }}{6}\left[ {\frac{{b_{{\text{s}}}^{2} \beta^{2} \delta_{{\text{t}}} }}{{b^{2} }}} \right.{ + }b\delta_{{\text{d}}} + \left. {2h\delta_{{\text{w}}} \left( {\beta^{2} - \beta + 1} \right)} \right]$$

The external load potential energy *V* of box girder under distortion loads is expressed as^24^

35 $$V = - \int_{0}^{l} {P_{{{\text{dt}}}} \gamma_{{\text{D}}} } h{\text{d}}z{ = } - \frac{b}{2}\int_{0}^{l} {\gamma_{{\text{D}}} P_{{\text{v}}} \left( z \right){\text{d}}z}$$

When disregarding shear deformation, the total potential energy $\Pi$ of the box girder under distortion loads can be expressed as $\Pi ={U}_{1}+{U}_{2}+V$. The requisite condition for attaining an extremum of $\Pi$ is that its first-order variation is equal to zero. Therefore, the governing distortion differential equation is derived as

36 $$\gamma_{{\text{D}}}^{\prime \prime \prime \prime } + 4\lambda_{{\text{E}}}^{{4}} \gamma_{{\text{D}}} = \frac{{\tilde{M}_{{{\text{DE}}}} }}{{EI_{{{\text{DE}}}} }}$$

where $\lambda_{{\text{E}}} = \sqrt[4]{{\frac{{EI_{{{\text{WE}}}} }}{{4EI_{{{\text{DE}}}} }}}}$ is the distortion attenuation coefficients, $EI_{{{\text{WE}}}} = 2K_{3}$ is the distortion frame stiffness, $\tilde{M}_{{{\text{DE}}}} = \frac{{P_{{\text{v}}} b}}{2}$ is the distortion external moment, and $EI_{{{\text{DE}}}} = 2H$ is distortion warping stiffness of the box girder, respectively.

Through a comparison of Eqs. 22, 30 and 36, it can be observed that the distortion geometric parameters of vertical web plate box girders, computed using the deformation coordination method, the plate element analysis method and the total potential energy variational method, satisfy the following relationships:

37 $$\lambda_{{\text{P}}} = \lambda_{{\text{E}}} = \lambda_{{\text{S}}}$$

38 $$EI_{{{\text{DP}}}} = 2EI_{{{\text{DE}}}} = 2EI_{{{\text{DS}}}}$$

39 $$EI_{{{\text{WP}}}} = 2EI_{{{\text{WE}}}} = 2EI_{{{\text{WS}}}}$$

40 $$\tilde{M}_{{{\text{DP}}}} = 2\tilde{M}_{{{\text{DE}}}} = {2}\tilde{M}_{{{\text{DS}}}}$$

It can be observed from Eqs. 37 ~ 40 that the distortion effects obtained by the three different methods are identical, demonstrating the consistency of the three different methods in calculating the distortion effects of a box girder.

Notwithstanding the notable variations in derivation processes and physical interpretations associated with these three methods for analyzing distortion effects, the distortion control differential equations generated by each approach exhibit complete consistency. This remarkable level of agreement holds significant theoretical significance.

First-order generalized beam theory (GBT) describes the behavior of prismatic structures by ordinary uncoupled differential equations, using deformation functions for extension, bending, torsion, and distortion. In the GBT theory, the ordinary differential equation is expressed as^9^

41 $$E{}^{k}C \cdot {}^{k}V^{\prime \prime \prime \prime } - G{}^{k}D \cdot {}^{k}V^{\prime \prime } + {}^{k}B \cdot {}^{k}V = {}^{k}q$$

where ^*k*^C, ^*k*^*D*, and ^*k*^*B* represent the section properties applicable to mode *k*. ^*k*^*V* represents the generalized deformation in mode *k*. ^*k*^*q* represents the distributed load applicable to mode *k*^10^. The theory of the deformation coordination method distortion effect of box beams can also be explained by generalized beam theory. The distortion differential equation obtained by the deformation coordination method also satisfies the GBT in which the warping constants satisfy the following conditions:

42 $$\begin{gathered} {}^{k}C = I_{{{\text{DS}}}} \hfill \\ {}^{k}B = I_{{{\text{WS}}}} \hfill \\ {}^{k}D = 0 \hfill \\ \end{gathered}$$

Vlasov’s thin-walled beam theory comes closest to GBT. Vlasov introduced the concepts of generalized coordinates and generalized displacements, enabling the determination of longitudinal and transverse displacements on the cross-section of box beams. Using the fundamental principles of elasticity theory, the strain and stress distributions in closed thin-walled box girders are determined. Subsequently, applying the principle of virtual displacements, a sixth-order differential equation with constant coefficients is derived to solve the restrained torsion problem of box girders with deformable cross-sections, as illustrated below.

43 $$f^{{{\text{VI}}}} - 2r^{2} f^{\prime \prime \prime \prime } + s^{4} f^{\prime \prime } = 0$$

Consequently, the restrained torsion problem incorporating distortion transforms into solving a sixth-order differential equation with constant coefficients for the function *f*(*z*). The generalized displacements and internal forces can be ascertained once the function *f*(z) is obtained. Unlike GBT, the generalized coordinate method does not decouple restrained torsion and distortion in box beams, considering only the shear stress generated by free torque while neglecting shear stress from constrained torsion in closed-section box beams. Moreover, Vlasov’s classical thin-walled beam theory applies only to doubly symmetric rectangular box beams. However, this theory remains an effective tool for analyzing the spatial force characteristics of box beams. This study adopts the fundamental principles of Vlasov’s generalized coordinate method, decoupling restrained torsion and distortion to separately analyze the distortion effects in box beams.

## Numerical examples

**Figure 6:**
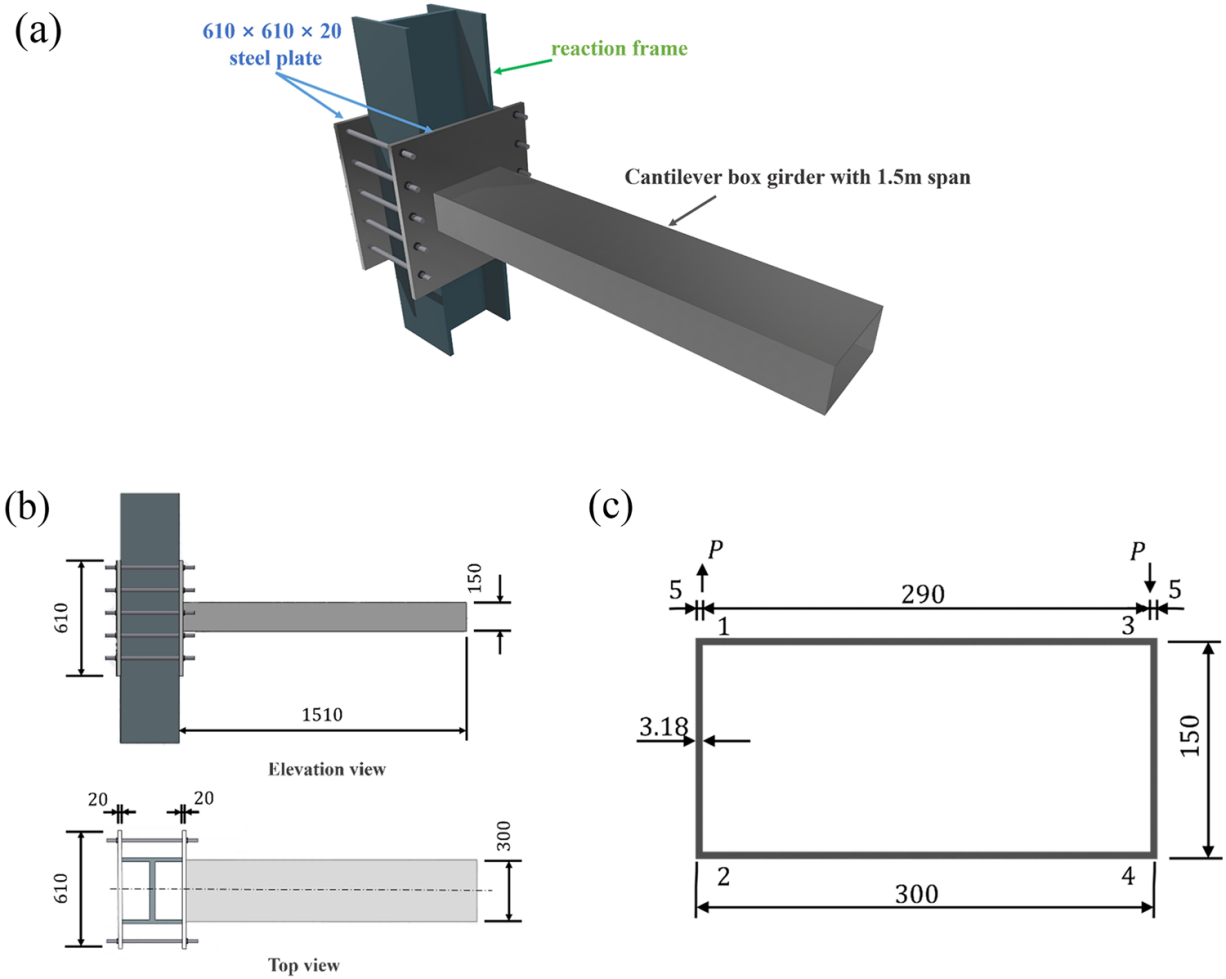
Configuration of cantilever box girder (unit: mm). (**a**)3D view (**b**) Layout of the cantilever box girder test (**c**) Cross-sectional dimensions and measurement point layout.

In the previous literature^17^, a bench scale test of a cantilever box girder was conducted to investigate the distortion effects. The specific configuration of tested cantilever box girder is shown in Fig. 6. The cross-section of girder is *b* × *h* = 300 mm × 150 mm, with a wall thickness of 3.18 mm. The employed cold-rolled low carbon steel plate with dimensions of 610 × 610 × 20 mm^3^ has an elastic modulus of *E* = 196.2 GPa. The selection of the measurement section, located at 3/4 of the span from the free end, was based on calculations that determined it to have minimal torsion-induced warping stress. Due to space limitations of the paper, the detailed calculations are not presented herein. Nonetheless, this choice ensures that the measured warping stress values obtained are representative of the overall behavior. The experiment utilized cold-rolled low-carbon steel plates with a thickness of 3.18 mm, which satisfies the essential assumptions of thin-walled box beam distortion theory, namely the insignificance of shear deformation and the uniform distribution of distortion stress across the wall thickness. It is important to note that challenges associated with boundary conditions and the accuracy of load application may introduce disparities between the experimental results and theoretical predictions. However, in accordance with Saint–Venant’s principle, the chosen test sections were specifically designed to effectively mitigate the influence of these adverse factors.

Table 1 tabulates the values of various distortion geometric parameters using the three different methods. The outcomes obtained through various methodologies fulfill the conditions stated in Eqs. 37 ~ 40, thereby confirming the correctness of the proposed formulas.

**Table 1:** Parameters associated with various theories employed for distortion calculation.

Plate element analysis method	Total potential energy variational method	Shear flow analysis method
$\gamma_{{\text{D}}}^{\prime \prime \prime \prime } + 4\lambda_{{\text{P}}}^{4} \gamma_{{\text{D}}} = \frac{{\tilde{M}_{{{\text{DP}}}} }}{{EI_{{{\text{DP}}}} }}$	$\gamma_{{\text{D}}}^{\prime \prime \prime \prime } + 4\lambda_{{\text{E}}}^{4} \gamma_{{\text{D}}} = \frac{{\tilde{M}_{{{\text{DE}}}} }}{{EI_{{{\text{DE}}}} }}$	$\gamma_{{\text{D}}}^{\prime \prime \prime \prime } { + 4}\lambda_{{\text{S}}}^{4} \gamma_{{\text{D}}} { = }\frac{{\tilde{M}_{{{\text{DS}}}} }}{{EI_{{{\text{DS}}}} }}$
$EI_{{{\text{DP}}}} = - \frac{{\Gamma_{{1}} b_{{\text{t}}} }}{{\Gamma_{{2}} }} = 10822{\text{ N}} \cdot {\text{m}}^{4}$	$EI_{{{\text{DE}}}} = 2H = 5411{\text{ N}} \cdot {\text{m}}^{4}$	$EI_{{{\text{DS}}}} = WA^{ * } = 5411{\text{ N}} \cdot {\text{m}}^{4}$
$\lambda_{{\text{P}}} = \sqrt[4]{{\frac{{EI_{{{\text{WP}}}} }}{{4EI_{{{\text{DP}}}} }}}} = 1.0729{\text{ m}}^{ - 1}$	$\lambda_{{\text{E}}} = \sqrt[4]{{\frac{{EI_{{{\text{WE}}}} }}{{4EI_{{{\text{DE}}}} }}}} = 1.0729{\text{ m}}^{ - 1}$	$\lambda_{{\text{S}}} { = }\sqrt[{4}]{{\frac{{EI_{{{\text{WS}}}} }}{{{4}EI_{{{\text{DS}}}} }}}} = 1.0729{\text{ m}}^{ - 1}$
$\tilde{M}_{{{\text{DP}}}} = P_{{\text{v}}} b = 1422.4{\text{ N}} \cdot {\text{m}}$	$\tilde{M}_{{{\text{DE}}}} = \frac{{P_{{\text{v}}} b}}{2} = 711.2{\text{ N}} \cdot {\text{m}}$	$\tilde{M}_{{{\text{DS}}}} = \frac{{P_{{\text{v}}} b}}{2} = 711.2{\text{ N}} \cdot {\text{m}}$

**Figure 7:**
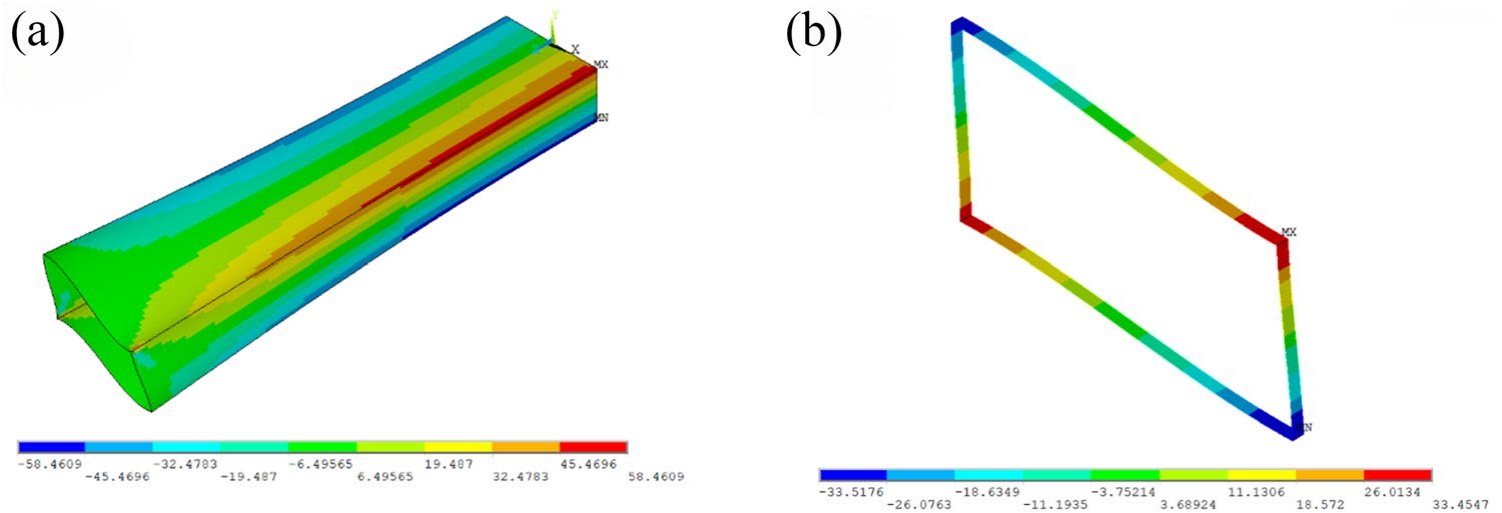
The normal stress contour diagram for the entire beam and the test section. (**a**) stress contour diagram for the entire beam and the test section. (**b**) stress contour diagram for the test section.

Furthermore, a finite element model is developed on the ANSYS software platform using the Shell 63 element. The finite element model employed in this study adopted a grid size of 10 mm, achieving the discretization of the box girder into 13,500 elements and 13,590 nodes. To ensure model coherence and stability, all nodal displacements at the fixed end were consistently constrained during the entire modeling process. Figure 7 illustrates the contour diagram depicting normal stress distribution for both the complete beam and the designated test section.

As shown in Table 2, the numerical results of the cantilever box girder are in good agreement with the experimental and analytical results with acceptable errors (< 10%), confirming the correctness of the proposed calculation methods in this paper. Table 2 demonstrates that the analytical and finite element solutions exhibit high agreement. Nonetheless, higher errors are observed at specific test points, primarily due to experimental challenges such as boundary conditions, load application accuracy, and sample preparation consistency. These factors may contribute to deviations between experimental results and theoretical predictions. Moreover, the precision and sensitivity of the sensors employed and the accuracy of data collection methods have a substantial impact on the final measurement outcomes.

**Table 2:** Distortion warping normal stress (unit: MPa).

Measurement point in Fig. 6	Analytical result ${\sigma }_{ANA}$	Finite element solution ${\sigma }_{FE}$	Experimental value ${\sigma }_{EXP}$	*Δ*_1_ (%)	*Δ*_2_ (%)
1	− 33.3	− 33.5	− 31.6	− 0.6	5.4
2	33.3	33.5	32.5	− 0.6	2.5
3	− 33.3	− 33.5	− 33.4	− 0.6	− 0.3
4	33.3	33.5	32.1	− 0.6	3.7

Δ_1_ = ($${\sigma }_{ANA}$$—$${\sigma }_{FE}$$)/$${\sigma }_{FE}$$ × 100%; Δ_2_ = ($${\sigma }_{ANA}$$—$${\sigma }_{EXP}$$)/$${\sigma }_{EXP}$$ × 100%

**Figure 8:**
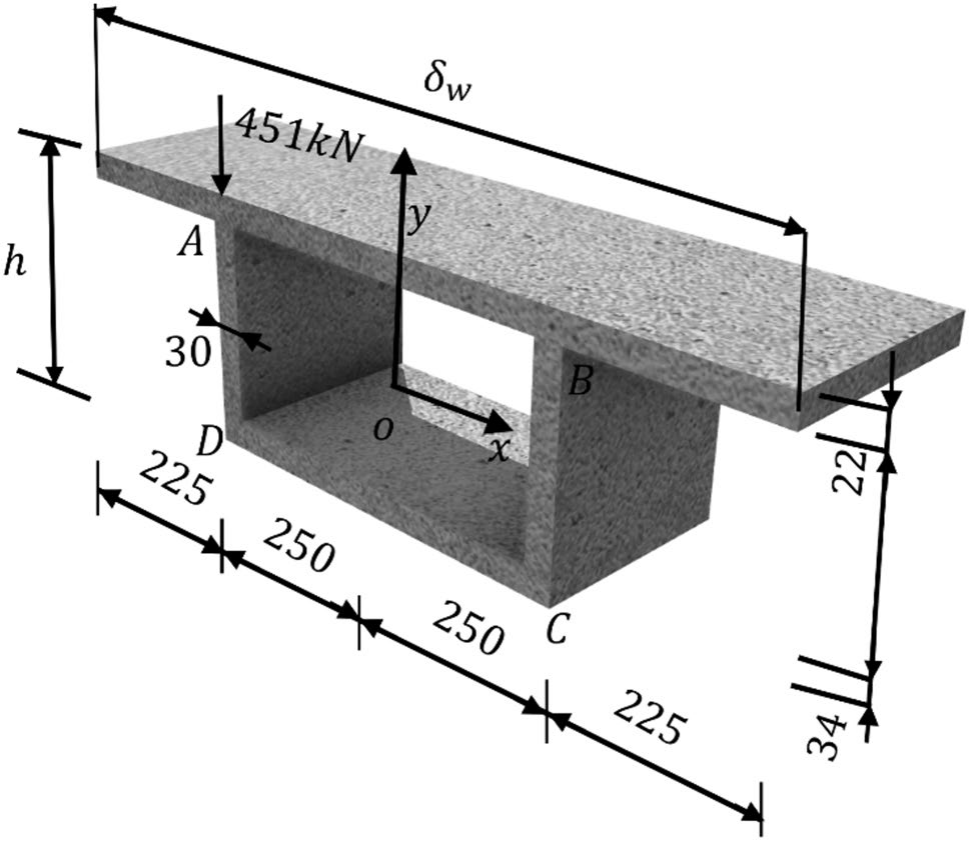
Simple support box girder.

In addition, a parametric study is conducted by taking a simply supported box girder bridge as an example. As shown in Fig. 8, the bridge span (*l*) is 40 m in length, the cross-section is 950 mm in width (${\delta }_{w}$) and 240 mm in height (*h*). The box girder is made of C40 concrete with an elastic modulus of *E* = 34 GPa. An eccentric load of P = 451.0 kN is applied at the top-left corner on the mid-span cross-section of the box girder.

**Figure 9:**
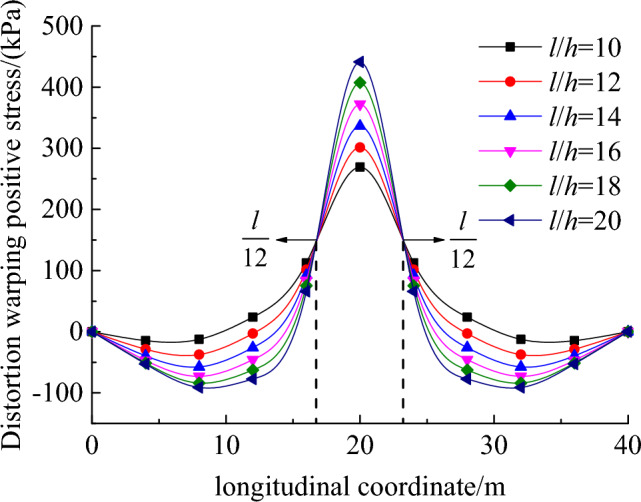
Effects of span-to-height ratio on the distortion warping normal stress distribution along the bridge span.

Figure 9 illustrates the variation of distortion warping normal stress at the loaded point with respect to the span-to-height ratio (*l*/*h*). It can be observed that the maximum distortion warping normal stress in the box girder occurs at the mid-span, and its peak value significantly increases with *l*/*h*. In contrast, the presence of stationary points at approximately *l*/12 from the mid-span of the box girder can be attributed to the support constraints imposed at both ends of the box beam. According to the stress distribution depicted in Fig. 9, the introduction of a diaphragm at the middle or quarter span in practical engineering applications can significantly alleviate the distortion effect encountered by the box beam.

**Figure 10:**
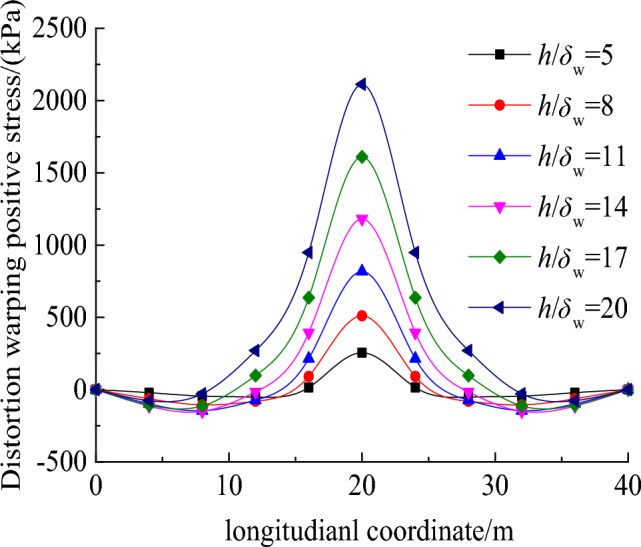
Effects of height-to-thickness ratio on the distortion warping normal stress distribution along the bridge span.

Figure 10 uncovers the variation of distortion warping normal stress at point *A* with the height-to-thickness ratio ($h$/${\delta }_{w}$). It can be observed that the peak distortion warping normal stress occurs at the mid-span and gradually decreases towards both ends of the beam. The distortion warping normal stress at the mid-span significantly increases with $h$/${\delta }_{w}$. Therefore, the increase in ${\delta }_{w}$ is an effective approach to reduce the distortion effects.

## Conclusion

This paper presents a novel deformation coordination method for analyzing the distortion effect of box beams. The method establishes a governing distortion differential equation to effectively control and mitigate distortion. A comparative analysis is conducted to evaluate the proposed method in comparison to the plate element analysis and the total potential energy variational method. Additionally, the study investigates the influence of geometric parameters on the distortion of box beams. Through comprehensive analysis, several major findings are concluded as follows:

1. In the case of vertically web-plated box girders, the governing distortion differential equation and distortion attenuation coefficients derived from the deformation coordination method presented in this paper align with the equations obtained through the plate element analysis method and the total potential energy variational method.
2. Under a concentrated load, the maximum distortion warping normal stress in the box girder occurs at the mid-span and increases with the span-to-height ratio. In contrast, at approximately 1/12 bridge span from the mid-span, the distortion warping normal stress remains constant regardless of variations in the span-to-height ratio.
3. As the height-to-thickness ratio of the web plate increases, there is a notable rise in the distortion warping normal stress at the mid-span cross-section. Consequently, enhancing the thickness of the box girder's walls emerges as an effective strategy for mitigating the distortion effects during the design phase of box girders.

Despite yielding fruitful outcomes, this study is subject to certain limitations. The analysis conducted solely investigates the distortion effects of box girders under simplified loading conditions. To explore the distortion effects under more intricate loading conditions, it is recommended to incorporate the nonlinear material properties of steel and concrete in the future research. Furthermore, while the present paper focuses exclusively on box girders, it is essential to extend the investigation to include other types of girders in order to verify the broader applicability of the proposed model. Nonetheless, this study provides valuable insights to engineers regarding the distortion effects on box girders.

## Data Availability

All the data presented in this manuscript are available upon request. For further inquiries and detailed information, please feel free to contact the first author Dr. Chenguang Wang at wcgcivil@mail.lzjtu.cn or the corresponding author Dr. Peng Wang at pwangal@connect.ust.hk.
